# Multiloculated omental cystic tumor hiding an angioleiomyoma: case report of a rare and atypical presentation and literature review

**DOI:** 10.1093/jscr/rjad231

**Published:** 2023-05-03

**Authors:** Mauro Giambusso, Paola Caprino, Franco Sacchetti, Angelo Eugenio Potenza, Dario Pastena, Luigi Sofo

**Affiliations:** Division of Abdominal Surgery, Department of Medical and Surgical Sciences, Fondazione Policlinico Universitario Agostino Gemelli IRCCS, Rome, Italy; Division of Abdominal Surgery, Department of Medical and Surgical Sciences, Fondazione Policlinico Universitario Agostino Gemelli IRCCS, Rome, Italy; Division of Abdominal Surgery, Department of Medical and Surgical Sciences, Fondazione Policlinico Universitario Agostino Gemelli IRCCS, Rome, Italy; Division of Abdominal Surgery, Department of Medical and Surgical Sciences, Fondazione Policlinico Universitario Agostino Gemelli IRCCS, Rome, Italy; Division of Abdominal Surgery, Department of Medical and Surgical Sciences, Fondazione Policlinico Universitario Agostino Gemelli IRCCS, Rome, Italy; Division of Abdominal Surgery, Department of Medical and Surgical Sciences, Fondazione Policlinico Universitario Agostino Gemelli IRCCS, Rome, Italy

## Abstract

Angioleiomyoma is a rare benign tumor arising from vascular smooth muscle and generally located in the subcutaneous tissue of the extremities. We reported a rare case of an intra-abdominal localization originating from the small omentum in which progressive growth detected on radiological follow-up indicated surgical excision. Histology documented a cavernous angioleiomuscular tumor with uncertain potential for malignancy. Although angioleiomyoma is described as a benign tumor, the uncertain behavior for malignancy of this case could have led to neoplastic degeneration. Early diagnosis followed by surgical excision of the neoplasia is crucial.

## INTRODUCTION

Angioleiomuscular tumors [1] are benign, solitary and vascular neoformations originating from smooth muscle cells of vessel’s *tunica media*. They are rare neoplasms, generally growing in the extremities, mainly the lower limbs.

Usually of incidental finding on imaging, only histological examination can confirm the diagnosis, as three phenotypes can be distinguished:

- solid: the most frequent, with compact smooth muscle cells and small, fissured vascular channels;

- venous: thicker, in which muscle tissue is more represented;

- cavernous: the rarest, with dilated vascular channels and few muscle cells.

Ultrasound and magnetic resonance imaging (MRI) are the imaging techniques that better identify the muscle-vascular structure of these lesions. On ultrasonography, features such as defined margins and a homogeneous structure of the neoplasia suggest its benign nature. High resistance in intratumor vessels suggests the presence of muscular arteries on color Doppler examination [2]. MRI findings include homogeneity and isointensity to skeletal muscle on T1-weighted images, whereas on T2-weighted scan these lesions appear predominantly isointense to surrounding fat but have extensive areas of internal linear and branching hyperintensity [3]. However, surgical excision followed by histological examination is still necessary to confirm diagnosis, with low recurrence rate [4].

## CASE REPORT

**Figure 1:**
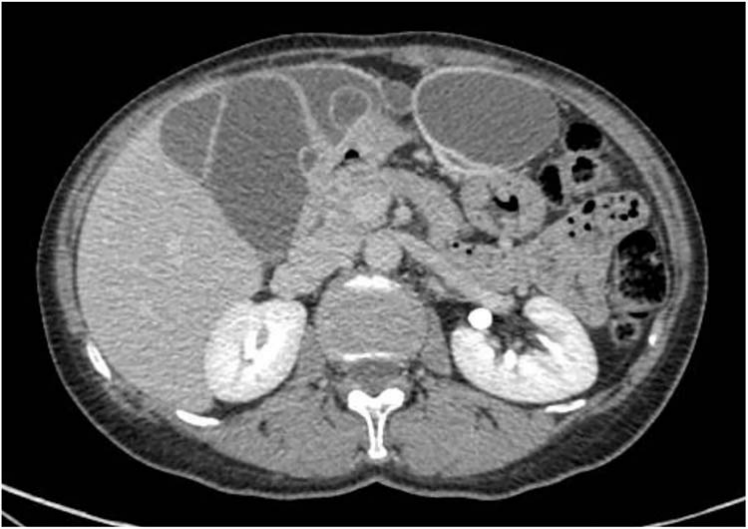
Axial CT-scan highlighting the abdominal pluriconcameral cystic formation.

**Figure 2:**
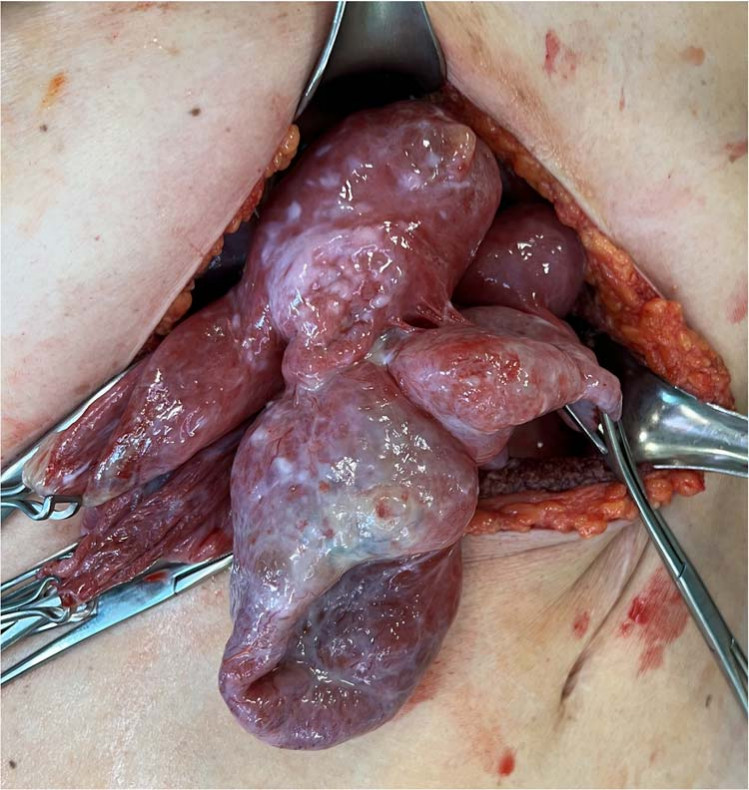
Intraoperative findings showing the multiloculated epigastric tumor.

**Figure 3:**
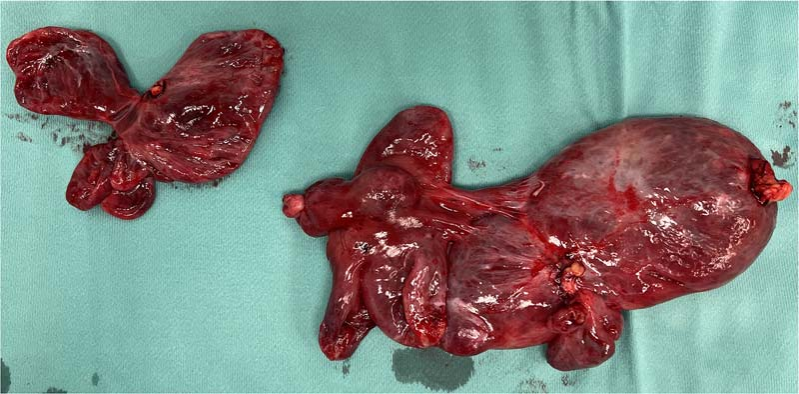
Surgical piece illustrating the two vascular pedicles of the neoformation.

A 59-year-old woman presented to the abdominal surgery unit of our hospital for a dimensional increase of known epigastric multiloculated cystic formation detected on computed tomography (CT)-scan follow-up, with a maximum diameter of~20 cm (Fig. 1). Her medical history included breast and colon adenocarcinoma and scapular melanoma, all of which were surgically treated in addition to chemo, hormone and radiation therapies. The patient was asymptomatic. Blood tests were unremarkable. At explorative laparotomy, a voluminous multi-chambered and well-vascularized cystic formation was found (Fig. 2). Surgical excision was performed after ligation of the two vascular peduncles located in the small omentum (Fig. 3). Histological findings showed an angioleiomuscular tumor with cavernous aspects and uncertain potential for malignancy. The patient was discharged on postoperative day 4 without complications. At follow-up visits at 1, 3 and 6 months, the patient was in good condition with no disease recurrence.

## DISCUSSION

Originally described by Stout in 1937 [5], angioleiomyomas are rare vascular neoplasms that arise from smooth muscle cells and generally affect the subcutaneous tissue, especially in the lower extremities. They are mainly localized in the hands [6] and feet [7, 8] as small, slow-growing, asymptomatic masses.

Our case describes an extremely rare type of angioleiomyoma originating from the small omentum. A similar case of close localization was reported by Qian *et al*. [9] in a patient with abdominal pain, where an exophytic mass arose from the portal vein. Abdominal involvement is rare in this pathology, which generally affects the gastrointestinal tract. The jejunum [10, 11] and ileum [12] are most commonly affected, manifesting themselves by episodes of melena. Although extremely rare, involvement of the larynx has been reported, which can enter the differential diagnosis with malignant tumors, as in the case published by Perardi *et al*. [13] in a patient with history of dysphagia and hoarseness.

If extremities represent the first localization of disease (about 90%), the second site is the head–neck district (10% of all cases) [14, 15]. Among these, the most frequent site of origin remains the oral region, as demonstrated by the review of Giudice *et al*. [16], with 63 cases described.

Other sites with more atypical presentations have been reported in literature, as the central nervous system (CNS), being able this pathology to occur throughout the body.

In our experience, angioleiomyoma diagnosis was hindered by the atypical radiological presentation, as a slow-growing multiloculated cystic neoformation with omental genesis. Moreover, the uncertain potential for malignancy, contrasting with the known benign nature of this condition, could have led to neoplastic degeneration. Surgical excision, indicated by volumetric growth, averted this fearful scenario.
